# Correction: Influence of variation in the volumetric moisture content of the substrate on irrigation efficiency in early potato varieties

**DOI:** 10.1371/journal.pone.0233407

**Published:** 2020-05-13

**Authors:** The PLOS ONE Staff

There is an error in affiliation 4 for author Izabela Kłosowicz. The correct affiliation 4 is: Prof. Bac’s Students Scientific Association of Melioration, Institute of Environmental Protection and Development, Wroclaw University of Environmental and Life Sciences, Wroclaw, Poland. The publisher apologizes for the error.
